# Use of Doxycycline in a Patient following Minocycline-Induced Lupus

**DOI:** 10.1155/2023/7353644

**Published:** 2023-07-05

**Authors:** Katherine Quinn Newman, Charles Guy Castles

**Affiliations:** ^1^Department of Medicine, Medical University of South Carolina, Charleston, SC, USA; ^2^Medical University of South Carolina, Charleston, SC, USA

## Abstract

Minocycline, a tetracycline antibiotic, is commonly used to treat rosacea and acne vulgaris. A rare adverse reaction of minocycline use is the development of drug-induced lupus. Fortunately, most patients recover from minocycline-induced lupus (MIL) after the drug is discontinued. However, many patients, after recovering from MIL, may desire further treatment for their acne and may consider doxycycline, a close relative of minocycline. Though no cases of doxycycline-induced lupus have been reported, there is little guidance in the medical literature as to whether doxycycline poses a particular risk to patients who have recovered from MIL. We report the long-term follow-up of a patient who recovered from MIL (the diagnosis satisfying clinical and laboratory criteria) and was treated for 8 years with various forms of doxycycline without any untoward effects, suggesting that, at least in some cases, doxycycline can be used safely following MIL.

## 1. Introduction

Drug-induced lupus (DIL) is a clinical syndrome which typically occurs in patients with no prior history of rheumatologic disease and consists of a combination of systemic symptoms along with autoantibody formation and is characterized by the prompt resolution of symptoms once the offending agent is discontinued [1]. Minocycline, a tetracycline antibiotic commonly used to treat acne vulgaris in young patients, is one of many drugs that have been associated with DIL. Because minocycline-induced lupus (MIL) often occurs in a young patient who is being treated for acne, after the minocycline is discontinued and the patient recovers, both physicians and patients may be interested in resuming treatment for their acne with another tetracycline. Though doxycycline has not, in contrast to minocycline, been associated with cases of DIL de novo, there is a paucity of literature, however, defining how doxycycline is tolerated in the particular group of patients who have recovered from MIL.

We report a case of a patient who was initially treated for acne with doxycycline for eleven months without adverse effects before switching to minocycline and eventually experiencing MIL. After discontinuing minocycline and completely recovering from MIL, she resumed treatment for acne with doxycycline and was followed for a period of eight years with no return of autoimmune symptoms or other untoward effects.

## 2. Case Report

The patient was a 15-year-old white female who began complaining of muscle and joint aches in November 2011, which were initially felt to be due to strenuous volleyball practices. Her past medical, family, and social history were noncontributory for autoimmune diseases, drug use, or significant illnesses. Over several weeks, her symptoms began to escalate to the point of interfering with school and she was noted to have elevated liver enzymes. She was referred to pediatric rheumatology at the Medical University of South Carolina (MUSC). While awaiting the appointment, her pediatrician stopped the minocycline which she had been taking for the previous 11 months for her acne. Her initial visit with rheumatology revealed an extensive polyarthritis involving her hands, wrists, and knees which subjectively had been improving after the discontinuation of the minocycline. Review of her laboratory abnormalities revealed a positive ANA, normal complement, a positive antidouble-stranded DNA, and positive antihistone antibodies. Her antihistone antibody level was 147 AU/mL (>120 AU/mL is positive). In addition, she had an elevated AST (44 U/L, range 14–36 U/L) and LDH (247 U/L, range 100−190 U/L) while her ALT (45 U/L, range 9−52 U/L) and GGT (20 U/L, range 12−43 U/L) were within normal limits. This could suggest her elevated AST and LDH were due to muscle involvement, though she never developed dark urine or elevated creatinine. Her rheumatoid factor was negative, and her C-reactive protein was normal (3.7, range < 5), though her IgG antibodies were elevated (1658, range 751–1560). The presumptive diagnosis of MIL was made. Though she was already improving after cessation of the minocycline, it was decided to prescribe her a short course of prednisone which she tolerated well. After tapering the prednisone, she transitioned briefly to nonsteroidal anti-inflammatory drugs (NSAIDs) and eventually stopped all medications. Within several months, she was completely back to her normal activities and abilities with no lasting effects. The patient had some labs repeated in May 2012, revealing normal liver enzymes, CBC, and urinalysis.

After full recovery, the patient desired to resume treatment for her acne. She tried a course of sulfamethoxazole/trimethoprim and azithromycin, before beginning her first round of isotretinoin (Claravis®, Barr Pharmaceuticals, Montvale, NJ) in February 2013. After two rounds of isotretinoin and still with unresolved acne, both she and her parents began looking for another treatment option and discussed using doxycycline. However, they were concerned that the literature was virtually silent on whether treating with doxycycline after MIL was advisable. It was decided to cautiously treat her with a form of doxycycline, Oracea® (Galderma, Lausanne, Switzerland). She underwent treatment for a year before stopping Oracea® and undergoing a third round of isotretinoin. During that time, she denied experiencing any of her previous symptoms. Doxycycline 40 mg (Orecea®, Galderma, Lausanne, Switzerland) was prescribed again in May 2017 and continued until February 2020. A generic doxycycline (50 mg/day) was also taken for one month in 2020. During the ensuing eight years after her recovery from MIL, she has had no rheumatologic or autoimmune symptoms. During this time span, she has undergone numerous treatments with doxycycline for acne, both generic and Oracea®, and experienced no untoward effects. It is also important to note that the patient had also taken Doryx® (Mayne Pharma, Greenville, NJ), a form of doxycycline, from October 2009 to January 2011, prior to taking minocycline. Doryx® had not caused any side effects or adverse events.

## 3. Discussion

Drug-induced lupus (DIL) is a syndrome characterized by a patient having at least one lupus-like feature, a positive ANA, and a temporal relation to ingestion of the offending drug. It occurs in patients without previous autoimmune disease, starting months to years after drug exposure, and subsides after cessation of the drug [2]. Some common symptoms of DIL are muscle and joint pain and swelling, fever, and even hepatitis, pleuritis, and pericarditis in some patients [3]. Sulfadiazine, in 1945, was the first drug reported to cause a lupus-like illness [4]. Since then, over 100 separate drugs and medications have been implicated in causing a lupus-like illness in patients. The most common medications associated with DIL are procainamide, hydralazine, isoniazid, quinidine, and minocycline and most recently the new biological immune modulators [1]. The drugs associated with DIL are classified by having a high, moderate, low, or very low risk for causing DIL [3].

Since drug-induced lupus rapidly resolves after stopping the inciting medication, differentiating systemic lupus from DIL is extremely important, though not always easy. To further complicate matters, some medications are felt to precipitate symptoms in patients with latent idiopathic SLE [1, 3]. Though clinically similar to systemic lupus (SLE), DIL tends to occur in slightly different populations, has slightly different laboratory and antibody features, and definitely has a different long-term prognosis. For instance, although all patients with SLE and DIL have autoantibodies, the occurrence and frequency of individual laboratory findings are quite variable, and there is no single definitive test to discriminate between DIL and SLE. Even among patients with DIL, variations exist depending on the inciting drug [2]. For instance, while a positive ANA is present in both systemic lupus patients and those with DIL, complement levels are frequently lowered in systemic lupus but are not involved in drug-induced lupus. In cases of DIL as opposed to idiopathic SLE, C-reactive protein (CRP) is almost always elevated. However, in the setting of serositis, CRP will most often be elevated for patients with both SLE and DIL [5]. Patients with DIL tend to be positive for antisingle-stranded DNA more so than antidouble-stranded DNA. In addition, antihistone antibodies occur in less than 50% of systemic lupus patients but occur in 95% of cases of DIL, with the interesting exception being MIL, where antihistone antibodies are uncommon, though they were positive in our patient. These differences suggest that DIL may have a pathogenesis separate and distinct from the mechanism of idiopathic systemic lupus. It is thought that the risk of developing DIL must depend on genetic and/or acquired differences in the metabolism of drugs [3]. A study by Dunphy et al. found that all 13 of their patients with MIL had either HLA-DR4 or HLA-DR2 [6, 7]. Also, because individual medications can cause different characteristics when precipitating DIL, it is conceivable that they may produce their effects via different mechanisms as well. It has been theorized that oxidative metabolites of the parent drug compound could play a role in triggering DIL [3, 7]. There have been many mechanisms for autoimmune induction suggested including nonspecific activation of lymphocytes by certain drugs, cytolytic potential of oxidative drug metabolites, disruption of immune tolerance, and the ability of drugs to act as haptens for T lymphocytes [3, 7].

Minocycline is a semisynthetic antibiotic in the tetracycline class and has both antibiotic and anti-inflammatory properties. It has been used for many years as a treatment for acne and is generally well tolerated. However, it has rarely been associated with a variety of autoimmune phenomena, including autoimmune hepatitis, serum sickness, vasculitis, and DIL [8]. It can also be associated with a life-threatening drug reaction, drug reaction with eosinophilia, and systemic symptoms (DRESS), which usually involves visceral organs such as the liver, kidneys, lung, and heart. Cardiac involvement is most commonly seen secondary to minocycline or allopurinol [8]. It presents frequently with dyspnea, hypotension, and/or chest pain and, unfortunately, has a high mortality rate.

Since the original case of MIL in 1992, MIL has become recognized as a rare association with this antibiotic [9]. We are reporting another case of a patient who developed MIL after having been treated with minocycline for acne vulgaris and are describing some of her unusual features and are reporting the successful use of doxycycline following recovery of MIL.

Our case had several unusual features, which, while not unique, are rare for MIL. For instance, elevated CRP is classically seen with DIL and, in fact, has been touted as a marker for DIL and consistently occurs with those rechallenged [10]. However, our patient had several CRP's drawn and all were within normal limits even during periods of obvious symptoms and active inflammation. This suggests that elevated CRP, while an important marker for possible MIL, should not be required to make the diagnosis. Second, unlike classic DIL seen with hydralazine and procainamide in which upwards of 75–95% of affected patients have positive antihistone antibodies, MIL is associated with antihistone antibodies with much less frequency [11–14]. The presence of antihistone antibodies in our case, though not unusual for DIL in general, is unusual for MIL and, like the normal CRP found in our patient, should be a reminder that not all patients present in the most classic manner.

Perhaps most importantly, since MIL tends to occur in young patients who are being treated for acne, once the minocycline is discontinued, some patients may want to resume some form of acne treatment and a natural option might be to try doxycycline. It is understood that the tetracyclines have different qualities and characteristics and, that minocycline in particular, is disposed to inducing a lupus-like syndrome [15, 16]. Large-scale studies have identified a rare but clear association between minocycline and lupus symptoms but have noted no such clear association with the other tetracyclines [15]. One report exists describing a patient who developed subacute cutaneous lupus after a short course of doxycycline, but the authors suggest that the doxycycline may have precipitated a flare up of a pre-existing subacute cutaneous lupus and actually represented a true case of drug induce lupus (ref). Despite these pharmacologic and statistical reassurances, only a few patients who have been diagnosed with MIL have been reported to have been subsequently treated with doxycycline, perhaps because knowledge of the overlapping biochemistry of the tetracyclines and the potential for cross-reactivity in other nonautoimmune conditions and likely due to the fear of the uncertainty of precipitating another bout of DIL [17].

Interestingly, though doxycycline, except possibly as described above, has not been implicated in cases of DIL de novo, there is a paucity of literature, however, defining how doxycycline might be tolerated in the particular group of patients who have been discovered to have MIL. One case has been previously reported in which a patient was given doxycycline six months after recovering from MIL and developed a recurrence of polyarthritis symptoms. These symptoms resolved once the medication was discontinued [18]. Two other patients, one with minocycline-induced pneumonitis and one with MIL, recovered after discontinuing their minocycline, and both were reported to tolerate subsequent use of doxycycline without return of symptoms [19, 20].

Minocycline, when used to treat acne, is often used for longer durations than other tetracyclines commonly used to treat acute infections. Because MIL typically occurs after several months (or longer) of treatment, one could argue that the potential recurrence of lupus symptoms may be tied to duration of treatment and perhaps if the doxycycline were to be used long enough it may eventually lead to problems. However, Lawson rechallenged 10 patients with previously diagnosed MIL and found that symptoms and elevated CRP recurred within hours of restarting minocycline [10]. Furthermore, our patient, after recovery of MIL, took several long courses of doxycycline over a period of several years with no recurrence of symptoms. Our patient is particularly interesting because she had taken one form of doxycycline, Doryx® (Mayne Pharma, Greenville, NJ), for over a year without symptoms prior to starting minocycline, suffered the MIL, recovered, and then took another version of doxycycline, Oracea® (Galderma, Lausanne, Switzerland) for several years without any symptoms.

It has also been suggested that some cases of DIL are actually idiopathic SLE that is “activated” by the offending medication [1, 3]. It is often difficult to distinguish between cases of DIL and “activated” SLE, and sometimes the correct diagnosis is only made after following patients for a while to see if they eventually develop SLE. Our patient has been followed for 8 years without the reappearance of other symptoms suggesting autoimmune disease, including after exposure to several courses of two forms of doxycycline. This length of follow-up is unusual for a case report and further supports the original diagnosis of MIL and thus the safe use of doxycycline in this setting.

In conclusion, we report an interesting case of a young girl who experienced MIL, recovered, and then was treated for eight years with doxycycline with no return of autoimmune or other symptoms. MIL may be uncommon but the desire to resume treatment for acne after recovery from MIL is also likely not rare. Though doxycycline, in contrast to minocycline, has not been definitively associated with DIL, the absence of case reports specifically documenting the safe use of doxycycline after recovery from MIL may still result in cautious hesitation by physicians and patients who might otherwise want to try doxycycline. Thus, it should be reassuring to physicians, pharmacists, and patients to know about cases that have been proven to do well on doxycycline after recovery from MIL. Though our case may not be representative of all patients with MIL, our patient demonstrates that several forms of doxycycline can be used for many years in some patients after MIL without precipitating a recurrence.

## Data Availability

No data were used to support this study.
